# In Vivo Optical Metabolic Imaging of Long-Chain Fatty Acid Uptake in Orthotopic Models of Triple-Negative Breast Cancer

**DOI:** 10.3390/cancers13010148

**Published:** 2021-01-05

**Authors:** Megan C. Madonna, Joy E. Duer, Joyce V. Lee, Jeremy Williams, Baris Avsaroglu, Caigang Zhu, Riley Deutsch, Roujia Wang, Brian T. Crouch, Matthew D. Hirschey, Andrei Goga, Nirmala Ramanujam

**Affiliations:** 1 Department of Biomedical Engineering, Duke University, Durham, NC 27708, USA; riley.deutsch@duke.edu (R.D.); roujia.wang@duke.edu (R.W.); brian.crouch@duke.edu (B.T.C.); nimmi.ramanujam@duke.edu (N.R.); 2 Department of Biology, Duke University, Durham, NC 27708, USA; jduer@wakehealth.edu; 3 Department of Cell and Tissue Biology, University of California, San Francisco (UCSF), San Francisco, CA 94143, USA; joyce.lee3@ucsf.edu (J.V.L.); jeremy.williams@ucsf.edu (J.W.); baris.avsaroglu@ucsf.edu (B.A.); andrei.goga@ucsf.edu (A.G.); 4 Department of Biomedical Engineering, University of Kentucky, Lexington, KY 40506, USA; caigang.zhu@uky.edu; 5 Duke Molecular Physiology Institute, Durham, NC 27701, USA; matthew.hirschey@duke.edu; 6 Department of Medicine, University of California, San Francisco, CA 94143, USA; 7 Helen Diller Family Comprehensive Cancer Center, University of California, San Francisco, CA 94143, USA; 8 Department of Pharmacology & Cancer Biology, School of Medicine, Duke University, Durham, NC 27708, USA

**Keywords:** fluorescence microscopy, fatty acid uptake, metabolism, breast murine tumor lines, metastatic potential, oncogene addition

## Abstract

**Simple Summary:**

A dysregulated metabolism is a hallmark of cancer. Once understood, tumor metabolic reprogramming can lead to targetable vulnerabilities, spurring the development of novel treatment strategies. Beyond the common observation that tumors rely heavily on glucose, building evidence indicates that a subset of tumors use lipids to maintain their proliferative or metastatic phenotype. This study developed an intra-vital microscopy method to quantify lipid uptake in breast cancer murine models using a fluorescently labeled palmitate molecule, Bodipy FL c16. This work highlights optical imaging’s ability to both measure metabolic endpoints non-destructively and repeatedly, as well as inform small animal metabolic phenotyping beyond in vivo optical imaging of breast cancer alone.

**Abstract:**

Targeting a tumor’s metabolic dependencies is a clinically actionable therapeutic approach; however, identifying subtypes of tumors likely to respond remains difficult. The use of lipids as a nutrient source is of particular importance, especially in breast cancer. Imaging techniques offer the opportunity to quantify nutrient use in preclinical tumor models to guide development of new drugs that restrict uptake or utilization of these nutrients. We describe a fast and dynamic approach to image fatty acid uptake in vivo and demonstrate its relevance to study both tumor metabolic reprogramming directly, as well as the effectiveness of drugs targeting lipid metabolism. Specifically, we developed a quantitative optical approach to spatially and longitudinally map the kinetics of long-chain fatty acid uptake in in vivo murine models of breast cancer using a fluorescently labeled palmitate molecule, Bodipy FL c16. We chose intra-vital microscopy of mammary tumor windows to validate our approach in two orthotopic breast cancer models: a MYC-overexpressing, transgenic, triple-negative breast cancer (TNBC) model and a murine model of the 4T1 family. Following injection, Bodipy FL c16 fluorescence increased and reached its maximum after approximately 30 min, with the signal remaining stable during the 30–80 min post-injection period. We used the fluorescence at 60 min (Bodipy_60_), the mid-point in the plateau region, as a summary parameter to quantify Bodipy FL c16 fluorescence in subsequent experiments. Using our imaging platform, we observed a two- to four-fold decrease in fatty acid uptake in response to the downregulation of the MYC oncogene, consistent with findings from in vitro metabolic assays. In contrast, our imaging studies report an increase in fatty acid uptake with tumor aggressiveness (6NR, 4T07, and 4T1), and uptake was significantly decreased after treatment with a fatty acid transport inhibitor, perphenazine, in both normal mammary pads and in the most aggressive 4T1 tumor model. Our approach fills an important gap between in vitro assays providing rich metabolic information at static time points and imaging approaches visualizing metabolism in whole organs at a reduced resolution.

## 1. Introduction

Cellular metabolism involves a vital network of pathways for homeostasis, growth, and survival. Alterations in these pathways are key features of a variety of diseases and conditions, such as diabetes, obesity, and cancer [1,2,3]. In cancer, oncogene activation or loss of tumor suppressors contributes to dysregulated metabolism, causing altered nutrient requirements compared to normal cells [4,5]. Aerobic glycolysis is frequently upregulated in tumor cells, and fluorodeoxyglucose-positron emission tomography (FDG-PET) can measure glucose uptake to stage a patient’s cancer, assess response to therapy, and evaluate progression [6]. Yet, a steadily growing number of studies suggest that tumors rely on additional energetic sources [7], including glutamine, amino acids, and lipids [8].

The use of lipids as a nutrient source is of particular importance in breast cancer, as an increase in lipid droplet formation is associated with increased tumor aggressiveness [9,10]. Adipocytes surround invasive breast cancers and serve as professional lipid storing cells, providing a readily available carbon source [11,12,13,14]. Adipocytes surrounding aggressive primary tumors show a loss of lipid content and increased IL-6 expression [15], a key inflammatory cytokine previously implicated in migration and invasion of breast cancer cells [16]. Further, fatty acids are often the preferential carbon source in drug-resistant and residual cancer [17,18,19].

Novel methods to quantify the use of new energetic sources, including lipids, in preclinical models will aid in the development of drugs to restrict uptake or utilization of these nutrients. In vitro and ex vivo assays, like the Seahorse Assay and metabolomics, quantify multiple metabolic endpoints; however, both are restricted to single, static time points and sacrifice spatial information. In vivo imaging technologies have the potential to allow for repeated measurements of single or multiple metabolic endpoint(s) for dynamically studying metabolic reprogramming. Further, imaging-based techniques could be used for studying the effectiveness of metabolically targeted therapeutics within the tumor microenvironment.

The ability to directly study fatty acid uptake within the context of an intact tumor-bearing animal is critical for evaluating novel therapeutics that antagonize lipid pathways or use lipids as an energy source. The most common in vivo metabolic imaging modality currently used for metabolic imaging is FDG-PET to measure glucose uptake with a radio-labeled glucose tracer [20]. Previous studies outside of breast cancer have validated the use of an additional radio-labeled PET tracer, 18F-fluoro-6-thia-heptadecanoic acid (18F-FTHA), for in vivo fatty acid uptake measurements for myocardium imaging [21], and, more recently, for prostate cancer [22] and melanoma [23]. Though metabolic imaging through PET has proved valuable, especially in the clinic, its millimeter-scale resolution prevents capture of tumor heterogeneity [24,25], which tend to be under 1 cm in diameter. Further, PET cannot be used to measure multiple endpoints at the same time.

Optical imaging allows for the temporal mapping of fatty acid uptake, as seen in PET imaging, but at a high enough resolution for spatial mapping of preclinical tumors, providing complementary metabolic information to existing techniques. Additionally, optical approaches have the capacity to image several endogenous sources of contrast [26] and can be coupled with appropriate exogenous indicators to provide functional and molecular information. In either case, high-resolution imaging of both in vitro and in vivo models of cancer can be performed [27,28,29,30,31,32,33]. Bodipy FL c16 (4,4-Difluoro-5,7-Dimethyl-4-Bora-3a,4a-Diaza-s-Indacene-3-Hexadecanoic Acid) is an exogenous indicator that has been successfully and extensively used in vitro to measure fatty acid uptake and, by extension, fatty acid (β) oxidation activity [34]. Bodipy FL c16 is comprised of a fluorophore and a 16-carbon long-chain fatty acid (palmitic acid), the most common saturated fat in animals [35]. Though Bodipy FL c16 has also been administered in vivo and quantified ex vivo by flow cytometry [36], optical in vivo imaging of Bodipy FL c16 to enable both longitudinal and spatial mapping of in vivo fatty acid uptake visualization has not previously been performed.

Here, we adapted and rigorously validated the fluorescent palmitate analog, Bodipy FL c16, for in vivo imaging of fatty acid uptake. For this study, we specifically developed our injection strategy and summary time points using intra-vital microscopy of a mammary window breast tumor model so as to visualize local changes in fatty acid uptake in the precise orthotopic region where the tumor was implanted; however, our approach can be adapted to optical imaging using a variety of technologies that differ in terms of depth of penetration, resolution, and field of view. Two different endpoints were used in our study: a summary parameter for overall Bodipy FL c16 uptake and a heterogeneity parameter providing spatial information. We performed intravital Bodipy FL c16 imaging of two syngeneic breast cancer models: the MMTV-Tet-O-MYC conditional model of triple negative breast cancer (TNBC) (MTB-TOM) [37] and the transplanTable 4T1, 4T07, and 67NR tumors in a mammary carcinoma model [38]. In the MTB-TOM model, Bodipy FL c16 fluorescence decreased following downregulation of MYC. Conversely, in animals with orthotopic transplantation of the 4T1 family of tumors [39], fatty acid uptake and heterogeneity increased with tumor metastatic potential. In this same 4T1 family orthotopic model, Bodipy FL c16 fluorescence was significantly decreased with the addition of a small molecule inhibitor, perphenazine, an inhibitor of fatty acid uptake [40], exemplifying another use of our approach: monitoring metabolic changes following therapy. In vivo optical imaging of fatty acid uptake with Bodipy FL c16 bridges an important gap in the field of cancer metabolism between in vitro-only single-time point assays, and in vivo PET imaging. Further, these methodology studies serve as the foundation upon which imaging of fatty acid uptake can be adapted to different types of optical imaging systems.

## 2. Results

### 2.1. In Vivo Imaging of Fluorescently Labeled Fatty Acids in Mammary Window Chambers

**Figure 1:**
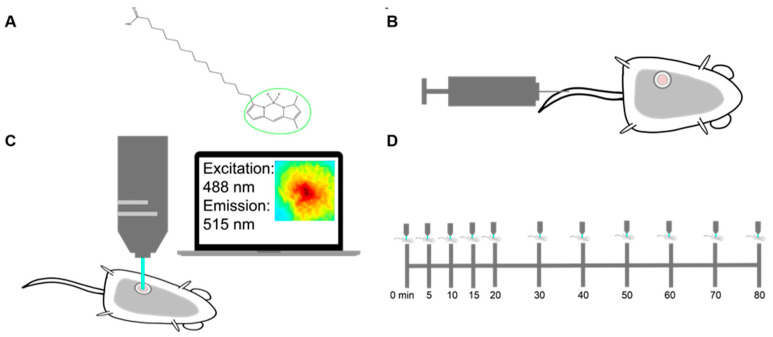
Methods for imaging palmitate uptake through Bodipy FL c16 in mammary window chambers using intravital fluorescence microscopy. (**A**) Schematic of fluorescently labeled palmitate, a 16-carbon-chain fatty acid (Bodipy FL c16). The fluorescent label is marked with a green circle. (**B**) Prior to imaging, a titanium mammary window chamber is implanted over the 4th right mammary fat pad of the mouse. 100 µL of 200 µM Bodipy FL c16 is administered via the tail vein for each imaging session. (**C**) Fluorescent probe accumulation into the mammary window chamber is measured by exciting the probe at 488 nm and collecting the emitted signal at 515 nm. (**D**) Timeline indicating the longitudinal imaging time course for each imaging session.

Due to their destructive nature, existing technologies that measure long-chain fatty acid uptake within tumor tissues are often limited in their ability to temporally study a single tumor [41,42]. Fluorescence imaging provides an opportunity to non-invasively study the dynamics of fatty acid metabolism over time, moving past the limitations of a static snapshot of fatty acid levels. To capture longitudinal fatty acid uptake and model a patient treatment cascade, we designed an imaging strategy using Bodipy FL c16. Bodipy FL c16, a commercially available fluorescent palmitate analog, consists of a 16-carbon long-chain fatty acid molecule linked to a Bodipy dye (Figure 1A). Bodipy FL c16 can be delivered systemically via tail vein injection (Figure 1B). Importantly, Bodipy FL c16 only enters cells via long-chain fatty acid transport proteins like normal fatty acids and not through simple cell membrane diffusion [34], making Bodipy FL c16 fluorescence a reliable indicator of true fatty acid transport. Once the probe is taken up into the target cell, it accumulates in the cytoplasm, providing an indicator of how rapidly cells are importing fatty acids. Because the 16th carbon of the palmitate is fluorescently labeled, immediate degradation of the Bodipy dye during fatty acid oxidation (β-oxidation) is prevented.

For intravital imaging, a 12 mm diameter titanium window was implanted over the tumor in the 4th mammary gland of each female mouse. The field of view within the window was excited at 488 ± 5 nm and collected at 515 ± 3.5 nm emission for fluorescence imaging (Figure 1C). To account for endogenous fluorescence differences between tumors, a background and dark image were collected and subtracted from each post-injection image to ensure all reported signals were only due to Bodipy FL c16. Each mouse was imaged sequentially for a total of 80 min post-Bodipy FL c16 injection (Figure 1D).

### 2.2. Bodipy FL c16 Uptake Differentiates MYC Oncogene Signaling in Tumors

Previous studies found that tumors that overexpress the MYC oncogene, such as receptor triple-negative breast cancers (TNBC), have increased uptake of stable isotope-labeled fatty acids in a MTB-TOM model [43]. We chose to assess our optical imaging technology using the same model, where MYC expression is activated by doxycycline (dox) [37,43]. We used a concentration of doxycycline (10 ng/mL) that allowed for the control of MYC expression without changes to fatty acid uptake levels, as shown in Figure S1, based on previous studies showing that doxycycline and other tetracyclines inhibit mitochondrial activity at and above the µg/mL level [44,45,46]. However, at the ng/mL level, the primary effect of doxycycline is in gene regulation [47,48,49]. Modulation of MYC through dox allows for temporal assessment of metabolism as tumors grow while on dox and regress after dox removal. To image Bodipy FL c16 uptake during murine tumor growth and regression, 100 µL of 200 µM Bodipy FL c16 was injected via the tail vein and fluorescence microscopy images (field of view: 2.1 mm × 1.6 mm) of the mammary tumor were captured over a span of 80 min post-injection while mice were receiving ad libitum dox (tumor growth with MYC-on) or four days after withdrawal of dox (early tumor regression with MYC-off [50]).

**Figure 2:**
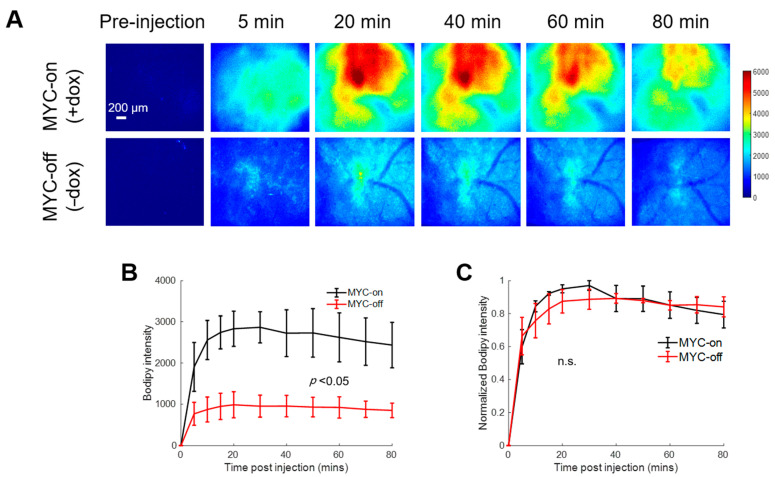
Bodipy FL c16 fluorescence peaks and then plateaus 30 min post-injection and MYC downregulation (MYC-off) results in a significant decrease in Bodipy FL c16 uptake. Bodipy FL c16 uptake was measured in mammary tumors overexpressing the oncogene MYC (+dox) and mammary tumors in which MYC was downregulated (−dox). (**A**) Representative images of Bodipy FL c16 fluorescence images over 80 min in a mammary tumor with MYC-on (+dox) and MYC-off (four days after dox was removed). Baseline images were acquired prior to Bodipy FL c16 injection and its average was subtracted from the images acquired after Bodipy FL c16 administration. Scale bar = 200 µm. (**B**) Average Bodipy FL c16 kinetic curves for the MYC-on (+dox) and MYC-off (−dox) groups. (**C**) For a given mouse, the kinetic curve was normalized to its peak Bodipy FL c16 fluorescence and normalized kinetics were then averaged for each group. Error bars represent standard error (sample size: MYC-on = 4 mice, MYC-off = 4 mice). The mid-point of the plateau, 60 min, was used to represent the Bodipy FL c16 (Bodipy_60_) fluorescence for subsequent analyses. Statistical differences in mean kinetic curves were determined using a two-way analysis of variance (ANOVA) test.

Figure 2A shows representative pre- and post-injection images from growing and regressing tumors, depicting the delivery kinetics of Bodipy FL c16 across the window chamber following Bodipy FL c16 systemic delivery in the MYC-on (+dox) and MYC-off tumors (−dox for 4 days). Fluorescence images are quantified via Bodipy FL c16 uptake curves in Figure 2B. Following MYC inhibition for 4 days, tumors regressed in size by ~2-fold (320 vs. 173 mm^3^; *p* < 0.05), and tumors’ Bodipy FL c16 uptake was significantly decreased, ~3-fold less at peak (Figure 2B; *p* < 0.05) compared to MYC-on tumors.

To illustrate that the delivery kinetics of Bodipy FL c16 were independent of Bodipy FL c16 uptake level, each mammary window’s kinetic curve in Figure 2B was normalized to its maximum fluorescence intensity. Normalizing each kinetic curve to its respective maximum fluorescence (Figure 2C) shows that, in both the MYC-on and MYC-off groups, Bodipy FL c16 accumulates into the window for approximately 30 min and remains stable for 30–80 min post-injection. We used the fluorescence at 60 min (Bodipy_60_), the mid-point in the plateau region, as a summary parameter to quantify Bodipy FL c16 fluorescence in subsequent experiments. Thus, MYC-dependent uptake of long-chain fatty acids can be visualized with Bodipy FL c16 in accordance with previously reported stable isotope uptake studies in this model [43].

### 2.3. MYC Overexpression Corresponds to Increased Fatty Acid Transport Protein Expression

**Figure 3:**
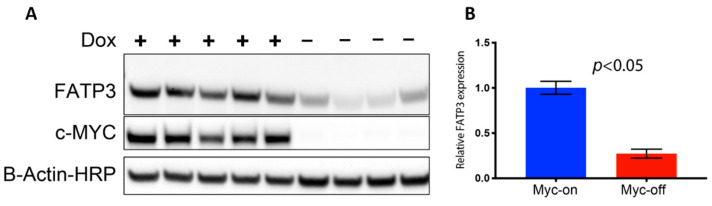
Loss of MYC expression results in decreased fatty acid transport protein 3 FATP3. (**A**) Western blots of FATP3 and MYC expression in tumors with (+) or without (−) dox for 4 days confirm FATP3 decreases as MYC expression decreases. Cropped Western blots are shown: full blots can be found in Figure S2. (**B**) Significant differences observed in FATP3 expression between MYC-on (+dox) vs. MYC-off (−dox) tumors. (Sample size: +dox = 5 mice, −dox = 4 mice). Statistical difference in expression was performed with a Student’s *t*-test.

Long-chain fatty acid (LCFA) uptake and trafficking within cells can be mediated by multiple receptors and carriers. Therefore, the increased Bodipy FL c16 uptake observed in MYC-on tumors may be mediated by multiple effector molecules. To evaluate which effector molecules are important for fatty acid uptake in MYC-driven tumors, we characterized the effect of MYC expression on cell surface transport proteins which have been reported to mediate LCFA uptake (Figure S2A). CD36 and the SLC27 gene family responsible for creating the fatty acid transport proteins (FATPs) have been shown to be critical in both the uptake and activation of long-chain fatty acids for fatty acid oxidation (β-oxidation) [51,52]. We analyzed RNA sequencing data of MTB-TOM tumors [53] to investigate how regulating MYC expression affects CD36 and FATP-family expression. When MYC was turned off through dox withdrawal, at an RNA level, *Slc27a3* was the mRNA sequence in the family that decreased in expression, though not significantly (Supplementary Figure S2A). However, MYC may indirectly regulate protein abundance by altering ribosome biogenesis and translation pathways [54,55,56]. The mRNA *Slc27a3* sequence specifically codes for FATP3; thus, we examined FATP3 protein expression in multiple MTB-TOM tumors. We found concomitant decreases in MYC and FATP3 protein expression when dox is removed during early tumor regression (Figure 3A; full blot, Supplementary Figure S2B). FATP3 protein expression was diminished by ~75% with the removal of dox (MYC-off) (Figure 3B; *p* < 0.05). While it is possible that multiple mechanisms contribute to increased Bodipy FL c16 uptake in MYC-on tumors, which are beyond the scope of the present study, we have identified at least one FATP-family member, FATP3, whose expression is correlated to MYC in a model of TNBC.

### 2.4. Longitudinal Imaging Reveals Modulation of MYC Expression Changes Mitochondrial Metabolism

**Figure 4:**
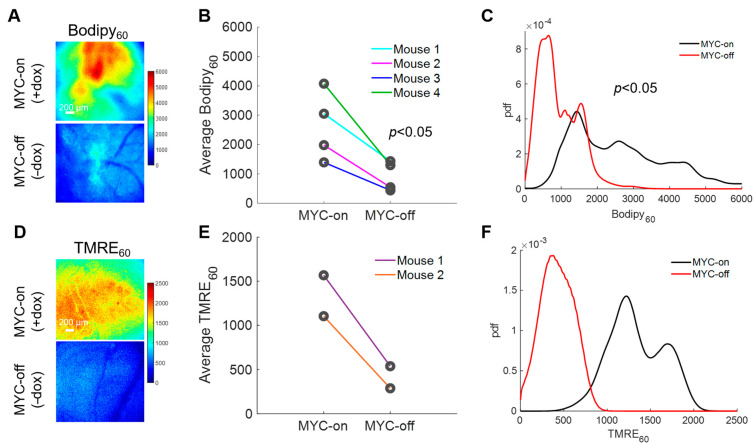
Intravital imaging demonstrates MYC dependence on fatty acid uptake and mitochondrial oxidation. (**A**) Representative Bodipy_60_ images for MYC-on (+dox) and MYC-off (−dox) tumors. (**B**) Average Bodipy_60_ fluorescence on day 0 (MYC-on) and day 4 (MYC-off) for four tumor-bearing mice. (**C**) Bodipy_60_ probability density functions (PDFs) for MYC-on (+dox) and MYC-off (−dox) across all pixels and all mice in each group. (**D**) Representative TMRE_60_ (TMRE fluorescence 60 min post-injection) images for MYC-on (+dox) and MYC-off (−dox). (**E**) Average TMRE_60_ fluorescence on day 0 (MYC-on) and day 4 (MYC-off) for two tumor-bearing mice. (**F**) TMRE_60_ probability density functions (PDFs) for MYC-on (+dox) and MYC-off (−dox) across all pixels and all mice in each group. Scale bar = 200 µm. Statistical differences in average fluorescence between groups were determined using a Wilcoxon signed-rank test, and statistical differences in fluorescent pixel distributions were determined using a Kolmogorov–Smirnov (KS) test.

With the importance of fatty acids in these MYC-on tumors established, we next explored how our method can longitudinally track metabolic changes in each animal following MYC downregulation. Longitudinal imaging was performed by imaging Bodipy FL c16 uptake in animals at baseline with MYC-on (+dox) and after MYC was turned off for four days (−dox for 4 days) (Figure 4A). Background images of the window chambers prior to the MYC-off imaging confirmed that Bodipy FL c16 had degraded, metabolized, or diffused out following the initial imaging session. We saw a significant decrease in average Bodipy_60_ fluorescence for MYC-off tumors compared to MYC-on tumors (*p* < 0.05) using a Wilcoxon signed-rank test (Figure 4B). Notably, we observed inter-tumoral heterogeneity in lipid accumulation but nevertheless saw each mouse’s individual decrease in Bodipy_60_ following MYC-downregulation (dox withdrawal). Additionally, probability density functions (PDFs), as shown in Figure 4C, were used to quantify the relative frequency of any given pixel intensity across all animals within an experimental group to see if inter-tumor heterogeneity masked changes in Bodipy FL c16 uptake. The PDF of MYC-off (−dox) is significantly left-shifted relative to the MYC-on (+dox) group by Kolmogorov–Smirnov (KS) testing (*p* < 0.05), signifying a decrease in the overall fluorescence (fatty acid uptake) in the tissue regardless of inter-tumor heterogeneity.

LCFAs serve as an important carbon source for oxidative phosphorylation after being broken down through β-oxidation in MYC-on tumors [43,57]; therefore, understanding how decreased availability of fatty acids through the reduction in MYC expression may affect oxidative phosphorylation would permit for dynamic in vivo assessment of tumor overall metabolic reprogramming. Previously, our group has established a method to optically measure oxidative phosphorylation in vivo in preclinical models via tetramethylrhodamine ethyl ester perchlorate (TMRE) [33]. This cationic molecule accumulates in proportion to the mitochondrial membrane potential [58], which serves as a surrogate for oxidative phosphorylation rates (i.e., increased mitochondrial membrane potential correlates with increased levels of oxidative phosphorylation). Due to the unknown optical, biological, or chemical crosstalk between Bodipy FL c16 and TMRE probes, a separate cohort of mice was used to investigate TMRE uptake in MYC-on and MYC-off tumors. Specifically, 100 µL of 75 µM TMRE was injected via the tail vein and fluorescence images were captured from the mammary window over a span of 60 min according to our previously validated methods [33]. We observed TMRE_60_ (TMRE fluorescence 60 min post-injection) images for MYC-on and MYC-off breast tumors (Figure 4D). Roughly a 3-fold decrease in TMRE_60_ fluorescence was observed following four days of MYC downregulation (Figure 4E). Additionally, the distribution of TMRE_60_ fluorescence shifted left in MYC-off (−dox) tumors relative to MYC-on (+dox) tumors, as shown by the PDFs in Figure 4F, indicating a decrease in oxidative phosphorylation. Here, we demonstrate the dynamic assessment of both LCFA uptake and oxidative phosphorylation in MYC-driven breast tumor models during tumor growth and early regression.

### 2.5. Using Bodipy FL c16 to Determine Drug Efficacy

We next demonstrated that Bodipy FL c16 imaging can be applied to study in vivo efficacy of drugs that attenuate LCFA uptake. We tested whether perphenazine, a drug previously shown to inhibit LCFA uptake [40,59], can reduce fatty acid uptake in 4T1 tumors, a highly metastatic triple-negative breast cancer cell line [39]. Perphenazine, a United States Food and Drug Administration (FDA)-approved drug used to treat patients with schizophrenia, binds to a subset of FATPs and reduces LCFA uptake in vitro and in a yeast-based functional screen [40,59].

**Figure 5:**
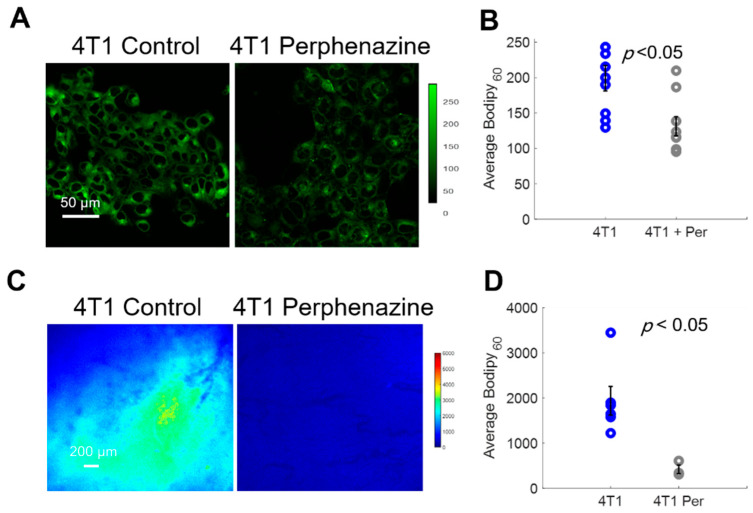
Bodipy_60_ decreases with the inhibition of fatty acid uptake. (**A**) Representative Bodipy Fl c16 images of 4T1 cells treated with PBS (4T1 control) or 80 µM of perphenazine (4T1 Perphenazine) for 2 h prior to staining with Bodipy Fl c16 for 30 min. Scale bar = 50 µm. (**B**) Average Bodipy FL c16 fluorescence per experimental group (sample size: all groups = 9 fields of view across 3 plates). (**C**) Representative Bodipy_60_ images of 4T1 tumors. Scale bar = 200 µm. (**D**) Average Bodipy_60_ fluorescence for each experimental group (sample size: Control = 6 mice, Perphenazine = 3 mice). Per = perphenazine-treated. Error bars = standard error. Statistical differences in average fluorescence between groups were determined using a Wilcoxon rank-sum test.

We first imaged Bodipy FL c16 uptake in vitro using a confocal microscope following a 2 h incubation with 80 µM perphenazine (Figure 5A) per standard protocols [40]. Bodipy FL c16 uptake was significantly decreased in 4T1 cells treated with perphenazine (Figure 5B) (*p* < 0.05). We also observed comparable results with two other cell lines from the same family (Figure S3). To monitor fatty acid uptake following perphenazine treatment in vivo, mammary window chambers were established, as previously described, over 4T1 tumors. On the day of imaging, the mammary window coverslip was removed and 80 µM perphenazine was topically applied to the exposed tissue for 2 h [40]. Following treatment, the window was rinsed with sterile phosphate-buffered saline (PBS), followed by our standard Bodipy FL c16 imaging protocol. Representative Bodipy_60_ images of a 4T1 tumor with and without perphenazine treatment are shown in Figure 5C. Short-term treatment with perphenazine significantly decreased Bodipy_60_ in 4T1 tumors (Figure 5D) (*p* < 0.05). We observed comparable results with in vivo non-tumorous mammary tissue treated with and without perphenazine (Figure S4).

### 2.6. Fatty Acid Uptake and Tumor Heterogeneity Increase with Metastatic Potential

Finally, because alterations in tumor metabolism can influence how aggressive a tumor is [9,10], we applied our Bodipy FL c16 imaging technique to test whether intravital Bodipy FL c16 imaging can distinguish between more and less aggressive tumors. The 4T1 tumor family, consisting of 67NR, 4T07, and 4T1 cell lines that arose from the same TNBC parental tumor [39], is known for the differing metastatic potential of each cell line [38]. The 4T1 tumor cells are considered the most aggressive because they have the highest rate of metastases, with secondary nodules often forming in the lungs and liver. The 4T07 tumor cells can mobilize from the primary tumor site but are only found in the blood, lymph nodes, and sometimes the lung, where they often fail to proliferate. The 67NR tumor cells are not detected in the blood, lymph nodes, or lungs [38,39]. Previous work has demonstrated that 4T1 and 4T07 cells have increased oxidative phosphorylation compared to 67NR cells as well as the 4T1 cells’ increased glycolytic flux compared to 67NR cells observed in an in vitro setting [60].

**Figure 6:**
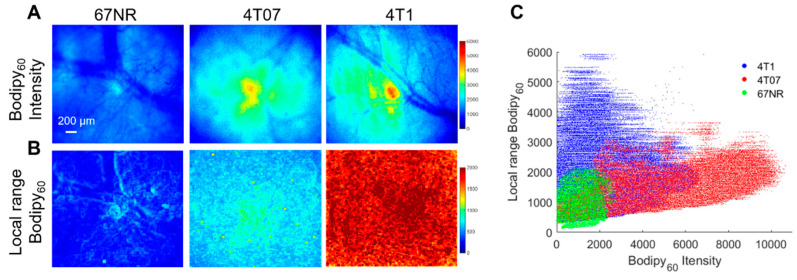
Bodipy_60_ intensity and heterogeneity is increased in metastatic-prone tumors. (**A**) Representative Bodipy_60_ intensity images of 67NR, 4T07, and 4T1 tumors in vivo. (**B**) Representative local range Bodipy_60_ images of 67NR, 4T07, and 4T1 tumors in vivo. Scale bar = 200 µm. (**C**) Bodipy_60_ fluorescence intensity vs. local range Bodipy_60_ for each pixel (sample size: 4T1 = 6 mice, 4T07 = 5 mice, 67NR = 5 mice). Statistical differences in average fluorescence intensity and local range between groups were determined using a KS test.

Both in vitro studies on cell monolayers and in vivo studies on mammary tumors in a window chamber model were performed here. Briefly, cell monolayers were incubated with 1 µM Bodipy FL c16 for 30 min prior to being washed and imaged immediately with a confocal microscope. Bodipy FL c16 uptake in 4T1 and 4T07 cell lines are significantly higher than that in the 67NR cell line (Figure S3). This trend is observed in vivo, as shown in Figure 6A. The 4T1, 4T07, and 67NR tumors were all grown to 5 × 5 mm in the mammary fat pad of female BALB/c mice, which maintain a fully intact immune system. Representative Bodipy_60_ images for each group are shown in Figure 6A. We also leveraged the spatial information within the images to investigate how tumor heterogeneity changes with metastatic potential. Local range images to represent heterogeneity, shown in Figure 6B, were created by calculating the local range (difference between maximum pixel intensity and minimum pixel intensity) for the nearest 1% of pixels for each respective pixel in an image.

The local range values for each pixel were plotted against its corresponding Bodipy_60_ intensity value in Figure 6C. The Bodipy_60_ intensity is increased in 4T07-bearing mammary window chambers compared to both 4T1- and 67NR-bearing windows, though only significant for 67NR (*p* < 0.05). We probed a recently published RNA-sequencing dataset and found that 4T1 cells have higher *Myc* mRNA when compared with 67NR cells [61]. While Slc27a3 mRNA expression followed the trend of *Myc* expression, it was not significantly different between 67NR and 4T1 cells (Figure S5). These data show that the more metastatic 4T1 and 4T07 primary tumors have increased fatty acid uptake compared to non-metastatic tumors. When looking at heterogeneity, both 4T1 and 4T07 tumors showed significantly increased local range relative to 67NR tumors (*p* < 0.05). 4T1 tumors also showed increased heterogeneity compared to 4T07 tumors that trended towards significant (*p* = 0.057).

## 3. Discussion

Our and others’ prior work indicate evidence of fatty acid dependencies in certain cancers [22,43,62,63,64,65,66]. However, there are surprisingly few technologies that can directly compare tumor lipid uptake in vivo, especially at length scales that can fill the gap between in vitro assays and whole-organ imaging. In the work presented here, we addressed this shortcoming in the literature through the development and validation of a fluorescence-based, longitudinal fatty acid uptake imaging approach in two distinct in vivo murine models. With this approach, we found increased fatty acid uptake in MYC-high tumors and tumors with high metastatic potential. Additionally, we demonstrate fatty acid inhibition with a reported decrease in fluorescence signal following the addition of perphenazine, a known fatty acid inhibitor. Finally, we demonstrate the utility of this imaging technique to capture fatty acid uptake heterogeneity in tumors, finding that the spatial heterogeneity of lipid metabolism is highest in the metastatic-prone 4T1 and 4T07 tumors compared to 67NR tumors, with the 4T1 tumors exhibiting the highest heterogeneity. Taken together, this allows for spatial and temporal reporting of cancer cell fatty acid uptake in local tumor microenvironments.

We tested our imaging approach in a MYC model, which has previously been shown to take up fatty acids from the circulation, validating the ability of our method to quantify fatty acid uptake in a regulated system. Bodipy FL c16 imaging, however, has the added advantage of permitting dynamic temporal imaging as MYC-driven tumors form and regress. The MTB-TOM model showcases two critically needed features for clinically relevant studies of fatty acid uptake: (1) longitudinal metabolite tracking in a single animal for intra-animal decreases in fatty acid uptake following oncogene downregulation, and (2) providing a link between fatty acid uptake and tumor aggressiveness.

Longitudinal imaging of Bodipy FL c16 led to the finding that MYC-dependent tumors have decreased fatty acid uptake following MYC downregulation, which serves as a proof of concept for future testing of MYC-specific therapeutic interventions. This decreased uptake is also correlated with a visible reduction in tumor size [50]. We link FATP3 expression with MYC expression. While our data suggest that treatment with inhibitors of FATP3 or knocking down this LCFA carrier protein would drive anti-tumor effects under the setting of MYC-high tumors, we did not directly test it here as it was beyond the scope of the present study. Future studies can be carried out to test the role of FATP3 in breast cancer. Indeed, studying Bodipy FL c16 uptake in MYC-driven tumors serves as an example of how potentially actionable targets can be identified when RNA-level and protein-level studies accompany optical imaging.

Intravital Bodipy FL c16 imaging can also report on the activity of fatty-acid-uptake-targeted therapies, such as perphenazine, both in vivo and in vitro. While we do not know the exact mechanism, perphenazine likely targets the FATP family. FATP (1–6) have roles in fatty acid uptake and/or activation in cells, so our group sought to image their effects on Bodipy FL c16′s uptake kinetics [59]. Prior research in yeast demonstrated perphenazine’s ability to block FATP2 [67]; however, there is likely some inhibitory effect on other FATP receptors. Our data supports future studies on perphenazine, where our method could be used to examine drug-dosing and minimal doses to target fatty acid uptake and oxidation in cancer, in order to reduce side effects. Though only one drug is tested here, the platform can be applied to a wide variety of fatty acid uptake or β oxidation inhibitors through either topical or systemic administration.

Previous studies have implicated increases in both fatty acid uptake and β oxidation in overall tumor aggressiveness and metastatic potential beyond MYC-inducible models [15,57,68,69,70,71]. The tumors of the 4T1 family of cell lines, which vary in metastatic potential (4T1 > 4T07 > 67NR), were selected to assess whether our technique could associate differences in fatty acid uptake across tumors of varying metastatic potential [39]. Bodipy FL c16 fluorescence was indicative of metastatic potential (higher in 4T1 and 4T07 vs. 67NR). Through an existing dataset, we show that *Myc* mRNA expression is higher in 4T1 cells in comparison with 67NR cells. This is consistent with previous findings that breast cancer patients with the highest *Myc* gene signature are most likely to have disease recurrence [72]. Aside from MYC-driven tumors alone, fatty acid oxidation in TNBC and its respective metastasis have recently been linked to the activation of the proto-oncogene tyrosine-protein kinase SRC [57]. SRC expression specifically has been shown to be elevated in both 4T1 and 4T07 tumors compared to 67NR tumors, and the knockdown of *Src* significantly inhibited the metastatic burden of 4T1 tumors [73,74]. The increased fatty acid uptake in all aggressive tumor lines suggests a metabolic switch associated with the use of exogenous fatty acids in the spread of metastatic disease. This idea is supported by previous studies implicating fatty acid uptake and oxidation as a key promoter of tumor cell migration [70,75,76].

Although 4T1 and 4T07 tumors have comparable Bodipy FL c16 fluorescence intensities, which are significantly higher than that of 67NR tumors, 4T1 tumors showed a greater degree of heterogeneity, measured by local range, compared to both 67NR and 4T07 tumors. Along with increased fatty acid metabolism reportedly correlated with increased metastatic and aggressive tumors, tumor metabolic heterogeneity has also been reported in the literature to correlate with increased tumor aggressiveness [75]. Previous work highlights the presence of lipid spatial heterogeneity, specifically, and the importance of capturing this metric to better phenotype the tumor metabolic landscape [76]. Further, increased heterogeneity captured via texture analysis has previously been associated with increased tumor grade and metastases [77,78]. Texture analysis has been extensively studied in clinical imaging in computed tomography (CT), PET, and (magnetic resonance imaging) MRI imaging and shows promise in improving diagnosis and tumor staging [79,80]. Here, we show that texture analysis of fatty acid uptake used in conjunction with the magnitude of the fluorescence intensity level can delineate highly metastatic (4T1) tumors from mildly metastatic (4T07) and non-metastatic (67NR) tumors. Because TNBC is a molecularly heterogeneous disease, imaging the tumor metabolic landscapes is vital to ensure a better understanding of a tumor’s energetic phenotype [81]. Future studies can apply this method to a broader group of tumor types that vary in metastatic potential to examine whether lipid uptake levels and heterogeneity throughout the tumor could be used as a complementary readout for reduced aggressiveness.

This work demonstrates that optical imaging of Bodipy FL c16 is important for lipid metabolism research. These studies are paramount to the successful translation of targeted metabolic therapies for TNBC and the plethora of other tumors that are dependent on fatty acids [57,63,82]. The drug screening protocol demonstrated here could also be applied to image the lung, liver, or brain through abdominal or cranial window chamber models, respectively [83,84]. Tumors in each of these locations have reportedly shown a dysregulated lipid metabolism [82,85,86]. More invasive window chambers of non-superficial organs, such as liver or lung windows, would require terminal imaging sessions; therefore, only single time points could be captured without more advanced surgical techniques. Additionally, the methodology developed here can be extended to other optical imaging modalities such as in vivo imaging systems (IVIS) or fluorescence spectroscopy systems for non-invasive monitoring of solid tumors. Collectively, this technique can provide a quantitative measurement for fatty acid uptake across various disease and imaging models to screen potential therapeutics.

In addition to the disease models used here, this technology can be used in cancer pharmacology research to quantify metabolic changes in human tumors following therapies through the use of patient-derived xenograft (PDX) models and organoids for prognostication and to inform future treatment regimens, as both have shown to recapitulate metabolic features seen in patients [87,88]. In fact, we have previously shown that exogenous fluorophores can be imaged using organoid models of human epidermal growth factor receptor 2 (HER2)-driven breast cancer [89]. Our method could also be useful for clinical applications, though the use of short-wavelength fluorescent indicators, such as Bodipy FL c16, would be limited to direct measurement of superficial lesions such as in the oral cavity, cervix, or skin, or imaging of biopsies taken from deeper seeded tumors. That being said, the methods showcased here can be adopted for a broader application with imaging modalities such as nuclear medicine (18F-FTHA) [22].

Optical imaging is complementary to currently available metabolic imaging methods, including positron emission tomography (PET) as well as magnetic resonance spectral imaging (MRSI). Each of these imaging approaches measures key metabolic endpoints by providing short-term uptake kinetics of labeled metabolites with differing tradeoffs. PET imaging, though most prevalently used for imaging glucose uptake, can also image fatty acids [22] and mitochondrial [90] metabolism, while MRSI can report on pyruvate and lactate uptake [91]. Both PET and MRSI can capture large fields of view, allowing for whole-organ imaging with relatively longer penetration depths than optical imaging, on the order of centimeters; however, increased penetration depth comes at the expense of decreasing resolution. Conversely, optical imaging can study similar metabolic pathways and endpoints across smaller length scales, providing the capability to image in vitro cell cultures or tumor window chambers at higher resolution, specifically on the order of microns, allowing optical metabolic imaging to fill a gap between in vitro cell-level assays and whole organ imaging.

We provide evidence supporting the use of fatty acid uptake with other validated metabolic imaging techniques (TMRE) to indicate effects on fatty acid oxidation in these tumors through the addition of mitochondrial membrane potential imaging [33]. These results are consistent with our prior work [43]. MYC overexpression can reprogram tumor metabolism in multiple ways, including uptake and utilization of multiple metabolites (e.g., LCFA, glucose, and glutamine [92]) that can contribute to increased oxidative phosphorylation. Through in vivo optical imaging, metabolic phenotyping of MYC-driven cancers is feasible. In addition, fluorescence imaging can capture multiple endpoints through spectral unmixing, as we have shown previously, where membrane potential and glucose uptake can be reported in the same imaging session [93]. The ability to capture multiple endpoints in vivo highlights the benefit of optical imaging as an important method for metabolically phenotyping cancer.

## 4. Materials and Methods

### 4.1. Ethics Statement

All in vivo murine experiments were conducted according to the Duke University Institutional Animal Care and Use Committee (IACUC) (Protocol A072-18-03). All mice were housed in an on-site housing facility with ad libitum access to food and water with standard light/dark cycles. Tumor volumes were measured using calipers and calculated as Volume = (Length × Width^2^)/2, where width represents the smallest axis and length the longest axis.

### 4.2. MYC-Overexpressing Murine Model

Viably frozen MTB-TOM tumors, generated from MTB-TOM (MMTV-rtTA/TetO-MYC) [37] (University of California, San Francisco, CA, USA), were divided into 1–2 mm chunks, rinsed in Dulbecco’s Modified Eagle Medium DMEM (Gibco, Montgomery Count, Maryland, USA) supplemented with 10 ng/mL doxycycline, 10 µm/mL insulin, and 50 µg/mL gentamycin, and transplanted into the 4th right mammary fat pad of 4-week old Friend Virus B (FVB/N) mice (Taconic, Rensselaer, NY, USA). Mice were administered 2 mg/mL doxycycline (Research Products International, Mount Prospect, IL, USA) through their drinking water, to maintain MYC overexpression and tumorigenesis. As tumors reached ~300 mm^3^, mice were imaged, and doxycycline was removed to initiate oncogene downregulation and tumor regression for 4 days prior to the second imaging session as tumors shrunk by roughly 50% in size. Four FVB/N mice were used for Bodipy imaging, while two FVB/N mice were used for TMRE imaging.

### 4.3. T1 Murine Model

The 4T1 and 4T07 cells were acquired from the American Type Culture Collection, and the 67NR cells were generously provided by Dr. Fred Miller (Karmanos Cancer Institute, Detroit, MI, USA) through Dr. Inna Serganova and Dr. Jason Koucher (Memorial Sloan Kettering Cancer Center, New York, NY, USA). Cell lines were passaged every 2 days in Roswell Park Memorial Institute (RPMI)-1640 medium (L-glutamine) with 10% fetal bovine serum (FBS) and 1% antibiotics (Penicillin-Streptomycin). For in vivo injection, the abdomen of 6–8 week old female BALB/c mice (Charles River) was cleaned using 70% ethanol and the 4th right mammary gland was palpated and injected with 100 μL (~50,000 cells) of 4T1 (*n* = 6 mice), 4T07 (*n* = 5 mice), or 67NR (*n* = 5 mice) cells suspended in sterile RPMI-1640 (Corning, Corning, NY, USA) with no FBS or antibiotics using a 27G needle. Tumors were imaged at a volume of ~150 mm^3^.

### 4.4. RNA Sequencing

The differential expression data from RNA-sequencing on the MTB-TOM tumors and 4T1 and 67NR cell lines were downloaded from GEO (GSE130922 and GSE104765) [53,61]. Data is reported as Log2FC after DESeq2 analysis [94] in R software.

### 4.5. Western Blot

Tumor tissue samples were flash-frozen in liquid nitrogen, and protein was extracted via a Dounce homogenizer in a cocktail of RIPA buffer (Thermo, Waltham, WA, USA) and proteinase (Roche, San Francisco, CA, USA) supplemented with phosphatase (Roche) inhibitor. Protein samples were resolved using 4–12% sodium dodecyl sulfate polyacrylamide gel electrophoresis (SDS-PAGE) gels (Life Technologies, Carlsbad, CA, USA) and transferred to nitrocellulose membranes (iBlot 2, Life Technologies). To probe for FATP3 (12943-1-AP, Proteintech, Rosemont, IL, USA, 1:1000) and c-MYC (MYC) (ab32072, Abcam, Cambridge, MA, USA, 1:1000), membranes were first incubated in primary antibody overnight on a 4 °C shaker, then incubated with horseradish peroxidase (HRP)-conjugated secondary antibody. To probe for β-actin (actin), membranes were incubated for one hour at room temperature in horseradish peroxidase (HRP)-conjugated antibody (sc-47778 HRP, Santa Cruz, 1:10,000). Signals were visualized with enhanced chemiluminescence (ECL) (Bio-Rad, Hercules, CA, USA), and chemiluminescence was acquired via Bio-Rad ChemiDoc XRS+. Unsaturated band intensities were quantified using Fiji software.

### 4.6. Murine Mammary Window Chamber Model

Using a previously established procedure, titanium window chambers with 12 mm diameter, No. 2 glass coverslips, were surgically implanted over the 4th right mammary gland of 8–12 week old female BALB/c (Charles River) [95].

### 4.7. Imaging Probes

For in vitro imaging, Bodipy FL c16 (4,4-Difluoro-5,7-Dimethyl-4-Bora-3a,4a-Diaza-s-Indacene-3-Hexadecanoic Acid, Thermofisher) was diluted in sterile RPMI-1640 (Corning) to a final concentration of 1 µM and incubated in each cell plate for 30 min. Next, cells were washed and imaged immediately with a standard confocal microscope (Zeiss 780 Upright confocal microscope at the Duke University Light Microscopy Core Facility). A total of 9 representative fields of view across 3 independent plates per cell line were imaged, and each cell line was measured on at least two different days.

For in vivo administration, Bodipy FL c16 was diluted to a final concentration of 200 µM in dimethyl sulfoxide (DMSO). This concentration was titrated to achieve a final tissue-level concentration (42.1–749.9 nM for these studies, calculated from tissue-mimicking phantoms (not shown)) below levels where self-quenching has been observed [96] and similar to in vitro studies [34]. TMRE (Tetramethylrhodamine Ethyl Ester, Life Technologies/ThermoFisher) was diluted to a final concentration of 75 μM in sterile PBS according to our previously established protocols [93]. Both probes were delivered systemically via tail vein injection, and the total volume of each injection was 100 μL.

### 4.8. Fluorescence Microscopy System and Metabolic Imaging

All mice were fasted for 4 h (water provided) prior to imaging to ensure a normalized metabolic rate for each animal [97]. Following fasting, imaging began immediately. The animal was anesthetized using isoflurane (1–1.5% *v*/*v*) in room air in an induction chamber. The animal was transferred to a heated (to maintain core body temperature) microscope stage where a background image (laser on) and dark image (laser off) were acquired prior to probe administration to account for signals from sources other than the fluorescence probes. Each animal received 100 µL of 200 µM Bodipy FL c16 in DMSO or 75 µM of TMRE in sterile PBS via tail vein injection and fluorescence images were acquired for 80 min. An exposure time of 5 s was used for both Bodipy FL c16 and TMRE images. To account for daily light source variation, all images were background subtracted, beam shape corrected, and calibrated according to a Rhodamine B standard imaged at each imaging session prior to image analysis. Each mouse was imaged only once. The only exception is with our genetically engineered mouse (GEM) model to track changes in metabolism on dox (MYC-on) compared to no dox (MYC-off), where each mouse was imaged twice, four days apart.

Our previously reported microscope [93] was used to capture fluorescence for both of our metabolic endpoints. For Bodipy FL c16 imaging, a 488 nm crystal laser (DL488–100-O, Crystal laser, Reno, NV, USA) was used to excite the probe, followed by a 505 nm long-pass dichroic mirror (DMLP505R, Thorlab, Newton, NJ, USA) to reject the excitation light in the emission channel. Emission signal was collected using a liquid crystal tunable filter (LCTF) (VariSpec VIS-7-35, PerkinElmer, Inc. Waltham, MA, USA, 7 nm bandwidth) programmed to collect at 515 nm and a high-resolution dual-modal charge-coupled device (CCD) (ORCA-Flash4.0, Hamamatsu, Japan). For TMRE imaging, a 555 nm crystal laser (CL555-100-O, Crystal laser, Reno, NV, USA) was used to excite the probe, followed by a 573 nm long-pass dichroic mirror (FF573-Di01-25 × 36, Semrock, Rochester, New York, NY, USA) to reject the excitation light in the emission channel. Emission signal was collected using the previously described LCTF (programmed to collect at 585 nm) and CCD. The spectral microscope system was calibrated wavelength by wavelength using a standard lamp source (OL 220 M, S/N: M-1048, Optronic Laboratories, USA).

This system uses a Nikon CFI E Plan Achromat 4× objective (NA = 0.1, Nikon Instruments Inc., Melville, NY, USA) for all imaging. This creates a single frame field of view of 2.1 × 1.6 mm and a lateral resolution of 2.2 µm, as measured using a 1951 USAF resolution target [93]. This microscope was controlled by a custom-designed LabVIEW software.

### 4.9. Fatty Acid Uptake Chemical Inhibition

For in vitro inhibition experiments with perphenazine, 4T1 cells were treated with 80 µM perphenazine (Sigma Aldrich, St. Louis, MO, USA) dissolved in sterile RPMI-1640 (Corning) for 2 h prior to in vitro staining and imaging according to the methods above. A total of 9 representative fields of view across 3 independent plates per condition were imaged, and each condition was measured on at least two different days. To ensure doxycycline does not interfere with Bodipy FL c16 uptake, 4T1 cells were treated with 10 ng/mL doxycycline dissolved in sterile RPMI-1640 (Corning, Corning, NY, USA) or PBS for 2 h prior to staining and imaged according to the protocol described above. A total of 9 representative fields of view across 3 independent plates per condition were imaged.

Separate cohorts of 4T1-bearing (*n* = 3 mice) or non-tumor (*n* = 3 mice) animals also received topical treatment with 80 µM perphenazine dissolved in sterile PBS. Following isoflurane anesthesia, the mammary window chamber was removed and 100 μL of perphenazine was applied to the exposed tissue. Following 2 h of incubation, sterile PBS was used to wash the window, the coverslip was returned to the tissue, and imaging began immediately according to the methods above.

### 4.10. Data Processing and Statistical Analysis

All post-processing and statistical analysis of Bodipy FL c16 fluorescent imaging was performed using MATLAB (MathWorks, Natick, MA, USA). Prior to further analysis, each image collected first underwent background and dark noise subtraction by removing the average value of an image collected with the laser source off (dark noise) and removing the average signal imaged prior to fluorescent probe injection (background). Additionally, due to the non-uniformity of illumination of a Gaussian light-source, each image was divided by an image of a uniform phantom to correct for the beam shape. Finally, each image was calibrated using a Rhodamine B standard solution imaged during each imaging session. These methods accounted for autofluorescence, non-uniform illumination, and day-to-day system variations, respectively. The resulting images were used for all statistical comparisons and images displayed in this study. The average intensity values at each imaging time point (0–80 min) were calculated to generate a time course kinetic curve. Comparisons of these curves were performed using a two-way analysis of variance (ANOVA). Additionally, comparison of average intensities using our summary parameter, Bodipy_60_, representing the fluorescence that is present 60 min post-injection, was performed using a Wilcoxon signed-rank test for paired time points in our MTB-TOM model and using a Wilcoxon rank-sum test followed by a post hoc Bonferroni correction for multiple comparisons in our 4T1 family of cell lines due to the non-normal distribution of Bodipy_60_ data points. Finally, local range images were quantified by calculating the local range of pixels representing 1% of the surrounding pixels for each respective pixel. Comparisons of all intensity and heterogeneity pixels for each group were performed using a KS test due to the non-normal distribution of pixels.

## 5. Conclusions

As the growing literature implicates fat as a key carbon source for many cancers, there is a need for a validated method to non-invasively image fatty acid uptake in vivo. In breast cancer alone, for example, exogenous fatty acid uptake and oxidation have been shown to be increased in primary TNBC [92], following therapy resistance in Her2 breast cancer [18], and in residual disease across breast cancer subtypes [19]. Pinpointing particular cancers with alterations in lipid metabolism may provide clues for targeting oncogenic metabolism in the clinic. We present a method for in vivo optical imaging of fatty acid uptake to metabolically phenotype in vivo tumors within their microenvironment. This technique is complementary to existing in vitro metabolic assays and in vivo PET and MRS imaging and can be used to detect and inform on alterations of tumor metabolism in preclinical studies and screen and validate potential therapy efficacy for translation to future clinical studies.

## Data Availability

The RNA sequencing datasets accessed for this manuscript are available in the publicly accessible repository, National Center for Biotechnology Information Gene Expression Omnibus (NCBI GEO). Gene expression values for MYC-driven tumors are available from GEO at https://www.ncbi.nlm.nih.gov/geo/query/acc.cgi?acc=GSE130922, GSE130922. Transcriptomic values of 4T1 and 67NR tumors are available from GEO at https://www.ncbi.nlm.nih.gov/geo/query/acc.cgi, GSE104765.

## Supplementary Materials

The following are available online at https://www.mdpi.com/2072-6694/13/1/148/s1, Figure S1: In vitro Bodipy FL c16 uptake with doxycycline treatment in 4T1 cells, Figure S2: RNA sequencing and full Western blot confirm removal of dox is correlated with decreased *SLC27a3*/FATP3 expression, Figure S3: Bodipy_60_ is increased in metastatic-prone tumors in vitro, Figure S4: Bodipy_60_ decreases with the inhibition of fatty acid uptake in normal, mammary gland tissue, Figure S5: RNA sequencing data analysis of murine breast cancer models.
