# Supplementary material for: In Vivo Optical Metabolic Imaging of Long-Chain Fatty Acid Uptake in Orthotopic Models of Triple-Negative Breast Cancer
Source: Cancers (Basel). 2021 Jan 5;13(1):148. doi: 10.3390/cancers13010148 (PMC7794847; doi:10.3390/cancers13010148)
Supplement: Supplementary file 1 [file cancers-13-00148-s001.pdf]

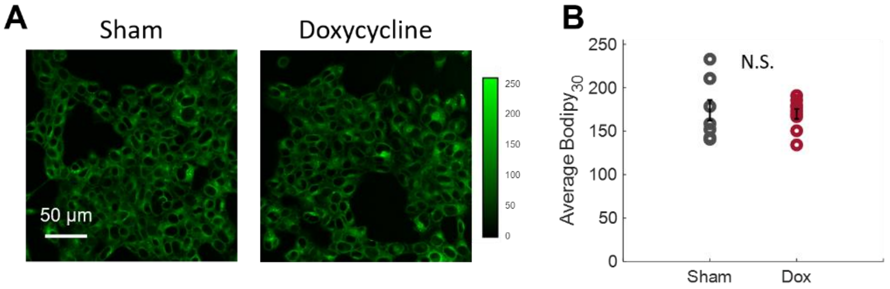

Figure S1. Doxycycline does not independently affect Bodipy FL c16 uptake in vitro. (**A**) Representative Bodipy Fl c16 images of 4T1 cells treated with PBS (Sham) or 10 ng/ml of doxycycline (Dox) for 2 h prior to staining with Bodipy Fl c16. Scale bar = 50 µm. (**B**) Average Bodipy FL c16 fluorescence per experimental group. (Sample size: all groups = 9 fields of view across 3 plates). Error bars = standard error. Statistical differences in average fluorescence between groups were determined using a Wilcoxon rank-sum test.

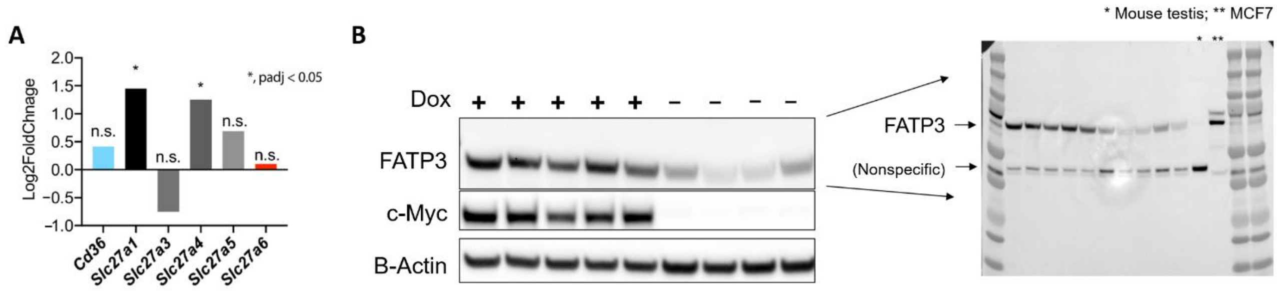

Figure S2. RNA sequencing and full western blot confirm removal of dox is correlated with decreased *SLC27a3*/FATP3 expression. (**A**) RNA sequencing data from MTB-TOM tumors comparing 4 days after dox withdrawal (regression) with MYC-on tumor. (Sample size: *n* = 3). (**B**) Western blots of FATP3 and MYC expression in tumors with (+) or without (−) dox for 4 days confirm FATP3 decreases as MYC expression decreases. (Sample size: +dox = 5 mice, −dox = 4 mice). Uncropped blots are shown to the right, with mouse testis (*) and breast cancer line MCF7 (**) as expression controls.

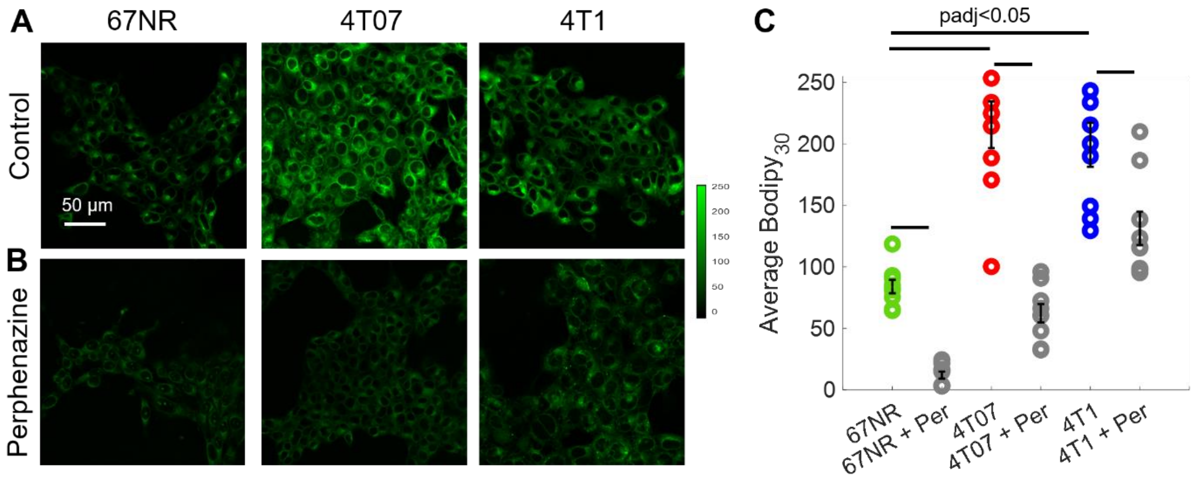

Figure S3. Bodipy$_{60}$ is increased in metastatic-prone tumors and decreased following perphenazine inhibition in vitro. Representative Bodipy Fl c16 images of 4T1, 4T07, and 67NR cells treated with (**A**) PBS (Control) or (**B**) 80 µM of perphenazine (Perphenazine) for 2 h prior to staining with Bodipy Fl c16 for 30 minutes. Scale bar = 50 µm. (**C**) Average Bodipy FL c16 fluorescence per experimental group. (Sample size: all groups = 9 fields of view across 3 plates). Error bars = standard error. Statistical differences in average fluorescence between groups were determined using a Wilcoxon rank-sum test followed by a post hoc Bonferroni correction for multiple comparisons.

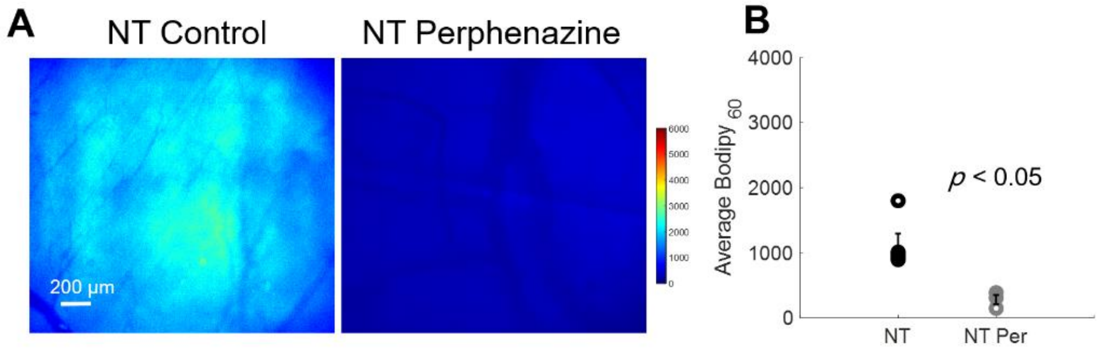

Figure S4. Bodipy60 decreases with the inhibition of fatty acid uptake in normal, mammary gland tissue. (**A**) Representative Bodipy60 images non-tumor (NT). Scale bar = 200 µm. (**B**) Average Bodipy60 fluorescence for each experimental group. (Sample size: Control = 5 mice, Perphenazine = 3 mice). Error bars = standard error. Statistical differences in average fluorescence between groups were determined using a Wilcoxon rank-sum test.

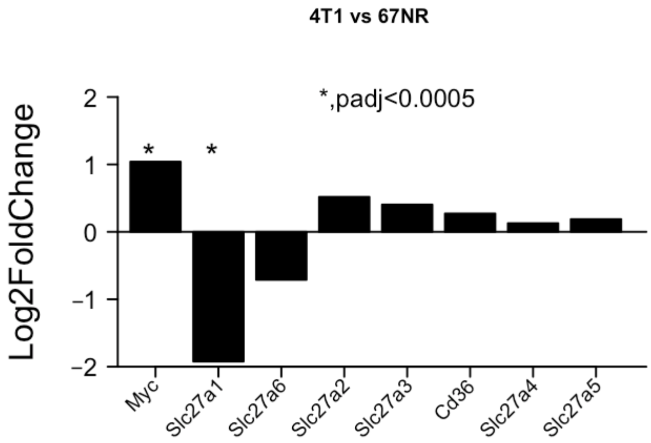

Figure S5. RNA sequencing data analysis of murine breast cancer models. 4T1 (highly metastatic) and 67NR (poorly metastatic) shows differential regulation of MYC. Here, an increased fold change corresponds with a gene expressed more in 4T1 cells than 67NR cells. (Sample size: $n = 3$).
